# Effect of bovine lactoferrin as a novel therapeutic agent in a rat model of sepsis-induced acute lung injury

**DOI:** 10.1186/s13568-019-0900-8

**Published:** 2019-10-31

**Authors:** Nannan Han, Hengjie Li, Gang Li, Ye Shen, Min Fei, Yong Nan

**Affiliations:** 1 Department of Emergency, Zhejiang Provincial People’s Hospital/People’s Hospital of Hangzhou Medical College, Hangzhou, 310014 Zhejiang China; 2 Health Examination Centre, Zhejiang Provincial People’s Hospital/People’s Hospital of Hangzhou Medical College, Hangzhou, 310014 Zhejiang China

**Keywords:** Bovine lactoferrin, Sepsis, Acute lung injury, Rats, Inflammation

## Abstract

Sepsis is a serious clinical condition resulting from severe infection. High rates of mortality and tissue damage have been reported in intensive care unit (ICU) patients with sepsis. Bovine lactoferrin (BLF) is a well-known 80-kDa glycoprotein in the transferrin family that inhibits sepsis in low-birth-weight neonates. The present study investigated the protective effects of BLF in a rat model of sepsis-induced acute lung injury (ALI). The wet/dry ratio, lipid peroxidation, antioxidant markers, total protein, total cell count, inflammatory markers and myeloperoxidase (MPO) levels were assessed. Histopathological analysis was also carried out. BLF treatment reduced the wet/dry ratio of lung tissue by 30.7% and 61.3%, and lipid peroxidation by 22.3% and 67%, at concentrations of 100 and 200 mg/kg, respectively. Superoxide dismutase (SOD), reduced glutathione (GSH), glutathione peroxidase (Gpx) and catalase were increased by more than 50% under treatment with 200 mg/kg BLF. Inflammatory markers, neutrophils, lymphocytes and total cell count were reduced by more than 50% under treatment with 200 mg/kg BLF. BLF treatment significantly reduced MPO activity, by 28.2% and 74.3%, at concentrations of 100 and 200 mg/kg, respectively. Neutrophilic infiltration and edema were observed in control rats. However, BLF treatment restored intestinal microvilli to the normal range and reduced inflammatory cell invasion. Collectively, these results suggest that BLF is an effective therapeutic agent against sepsis-induced ALI.

## Introduction

Sepsis is a serious clinical condition caused by severe infection (Taşcı et al. 2017). Researchers have reported that sepsis is associated with high rates of mortality and tissue damage in intensive care unit (ICU) patients (Baracchi et al. 2011). Sepsis leads to multiple organ failure and lung dysfunction (Fujishima 2016). Acute lung injury (ALI) is associated with tachypnea and hypoxemia (Randhawa and Bellingan 2007), and researchers have reported that acute respiratory distress syndrome (ARDS) is linked with ALI (Matthay et al. 2012). ALI is associated with high rates of morbidity and mortality (Fang et al. 2012), and is responsible for 74,500 deaths per year in the Western countries (Walkey et al. 2012). Increased permeability of the alveolar-capillary membrane, pulmonary edema, accumulation of protein-rich fluid in the airspaces, poor lung performance, and pulmonary infiltration of neutrophils are key symptoms of ALI (Matute-Bello et al. 2008). Favarin et al. (2013) reported that there is currently no cure for ALI, and sepsis increases its mortality rate. Thus, identifying an effective therapeutic agent is a high priority.

Bovine lactoferrin (BLF) is a well-known 80-kDa glycoprotein within the transferrin family. Manzoni et al. (2009) reported that BLF inhibits sepsis in low-birth-weight neonates. Similarly, Chen et al. (2014) demonstrated the therapeutic effect of aerosolized BLF on lung injury and fibrosis in mice. Cutone et al. (2019) reported that aerosolized BLF reduced infection, iron imbalance and inflammation in chronic lung infection, and Hegazy et al. (2016) demonstrated renoprotective effects of BLF in acute kidney injury. Aerosolized BLF reduced pro-inflammatory cytokines and neutrophils in a mouse model of lung infection (Valenti et al. 2017). Therefore, the current study investigated the protective effect of BLF against sepsis-induced ALI in a rat model.

## Materials and methods

### Rats

Rats were obtained from the animal house of Zhejiang Province People’s Hospital/People’s Hospital of Hangzhou Medical College, China. The rats weighed 190–210 g and rats were kept in standard rat polypropylene cages (435 × 290 × 150 mm; six rats per cage) and maintained under standard conditions of 12 h light/12 h dark at a relative humidity of 60 ± 5% and temperature of 25 ± 0.5 °C with food and water provided ad libitum. All rats were maintained under appropriate conditions according to ethical standards for animal welfare.

### ALI and rat groups

Experimental ALI was induced according to Filgueiras et al. (2012). Rats were divided into sham, control (ALI), ALI + 100 mg/kg body weight (bwt) BLF (L9507, Sigma-Aldrich, Shanghai, China), and 200 mg/kg bwt BLF groups. 24 h after ALI induction, rats were given BLF for 30 consecutive days through oral gavage. Sham and control rats were administered an equal volume of saline. At the end of the treatment, the blood and bronchoalveolar lavage fluid (BALF) were collected.

### Measurement of wet/dry ratio, lipid peroxidation and antioxidant markers

The wet/dry ratio of lung tissue by weight was determined according to Huang et al. (2017). The levels of lipid peroxidation in fresh lung tissue homogenates were determined according to Toufekoula et al. (2013). Serum levels of superoxide dismutase (SOD), reduced glutathione (GSH), glutathione peroxidase (Gpx) and catalase were determined according to Zhang et al. (2019).

### Determination of protein content and total cell count

In the BALF, the level of total protein was determined using the bicinchoninic acid (BCA) method according to Yalamati et al. (2015). Neutrophils, lymphocytes and total cells in the BALF were measured according to Domagała-Kulawik et al. (2012).

### Determination of inflammatory markers and myeloperoxidase

Migration inhibitory factor (MIF), interleukin-8 (IL-8) and tumor necrosis factor-alpha (TNF-α) levels were measured using ELISA assay kits according to a previously reported method (Kothari et al. 2013). Levels of myeloperoxidase (MPO) in the homogenate were measured according to a previously reported method (Queiroz-Junior et al. 2009).

### Histopathological study

Lung histopathological analysis was carried out according to a previously reported method (Althnaian et al. 2013). Briefly, the lower pulmonary lobes were immersed in 10% formalin and then embedded in paraffin. Then, 4-µm-thick sections were prepared and stained by hematoxylin and eosin (H&E). Lung tissue sections were examined under a light microscope.

### Statistical analysis

Experimental results are given as mean ± standard deviation (SD). All data were analyzed and compared using analysis of variance followed by Tukey’s post hoc test. A threshold of *P* < 0.05 was taken as significant.

## Results

### Effect of BLF on wet/dry ratio of lung tissue

**Fig. 1:**
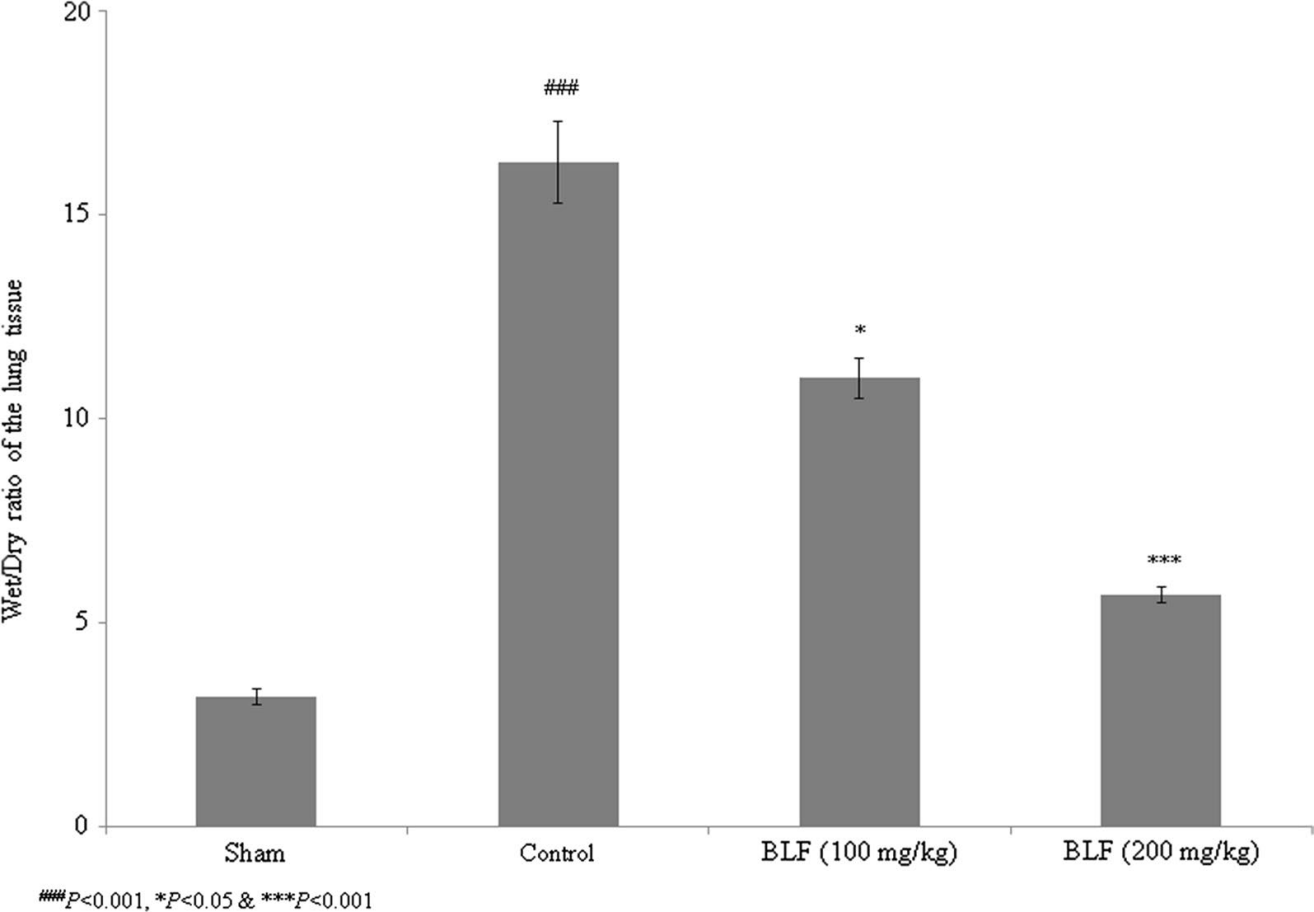
Bovine lactoferrin (BLF) reduces the wet/dry ratio of lung tissue in acute lung injury (ALI)-induced male albino rats. Rats were given BLF for 30 consecutive days through oral gavage. ^###^*P* < 0.001 compared to the sham group; *P < 0.05 and ***P < 0.001 compared to control rats

We observed protective effects of BLF against sepsis-induced ALI in the rat model. The wet/dry ratio of tissue increased by 406.2% in the control rats (Fig. 1, *P* < 0.05). However, BLF treatment decreased the wet/dry ratio of lung tissue by 30.7% and 61.3% at concentrations of 100 and 200 mg/kg, respectively (Fig. 1, *P *< 0.05).

### Effect of BLF on lipid peroxidation

**Fig. 2:**
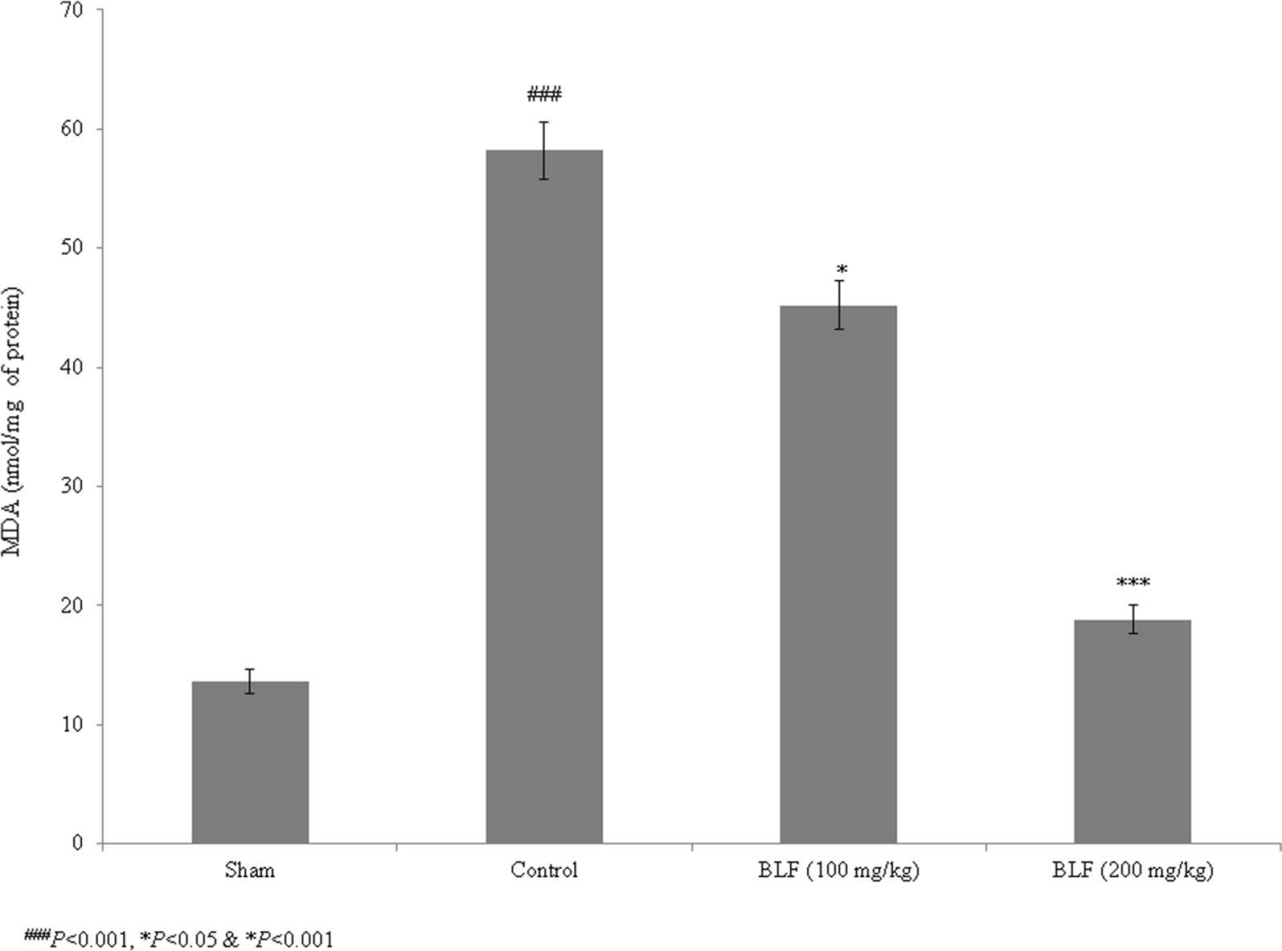
BLF reduces lipid peroxidation in lung tissue homogenate of ALI-induced male albino rats. Rats were given BLF for 30 consecutive days through oral gavage. ^###^*P* < 0.001 compared to the sham group; *P < 0.05 and ***P < 0.001 compared to control rats

Lipid peroxidation was determined based on malondialdehyde (MDA) levels in the lung tissue homogenate. Lipid peroxidation dramatically increased, by 323.5%, in the control rats compared to the sham rats. However, BLF treatment reduced the MDA content by 22.3% and 67% at concentrations of 100 and 200 mg/kg, respectively (Fig. 2, *P *< 0.05).

### Effect of BLF on antioxidants

**Fig. 3:**
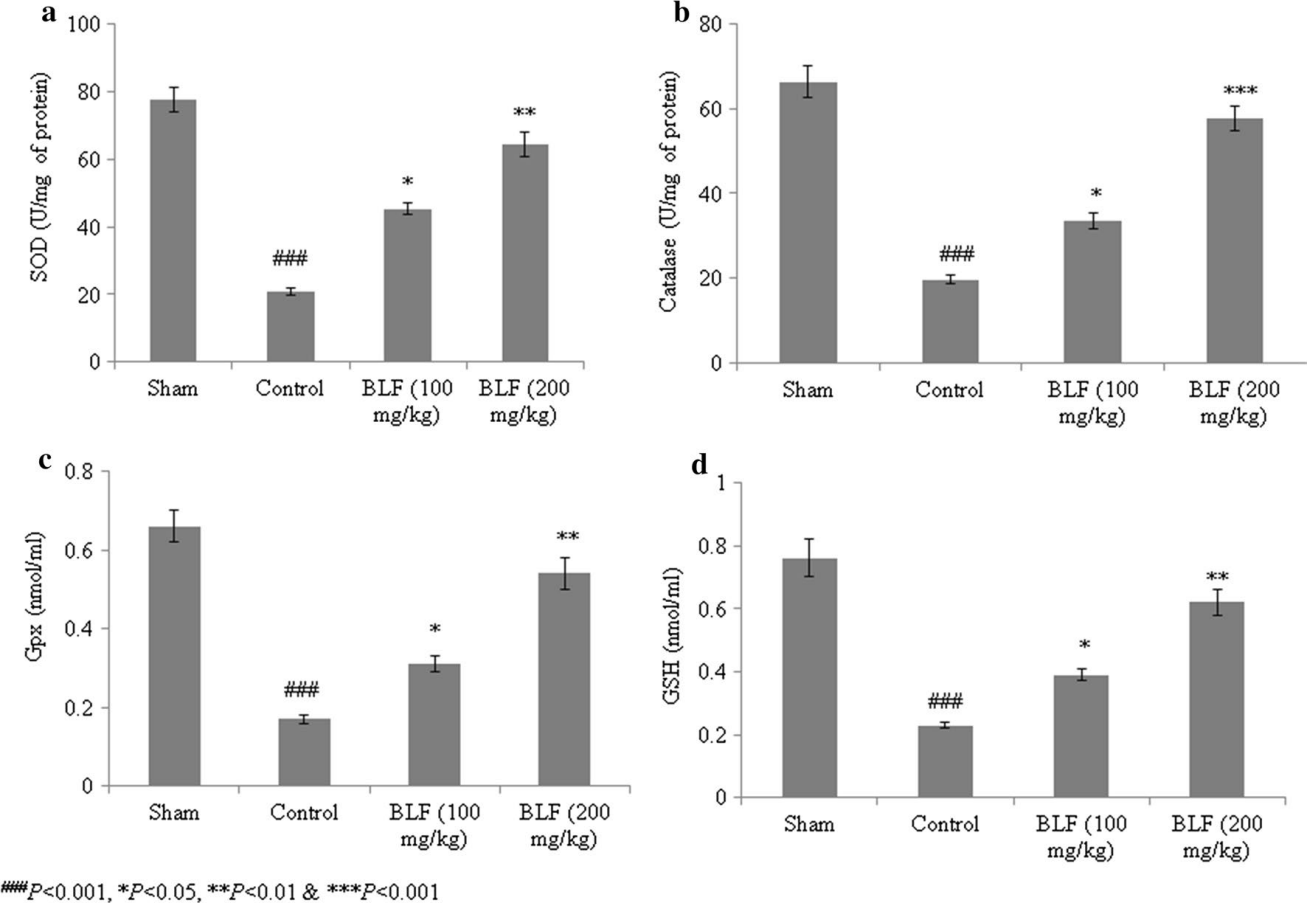
BLF increases superoxide dismutase (SOD) (**a**), catalase (**b**), glutathione peroxidase (Gpx) (**c**) and glutathione (GSH) (**d**) in the lung tissue homogenate of ALI-induced male albino rats. Rats were given BLF for 30 consecutive days through oral gavage. ^###^*P* < 0.001 compared to the sham group; *P < 0.05 and ***P < 0.001 compared to control rats

SOD, Gpx and catalase activity substantially decreased in the control rats. However, BLF treatment significantly increased SOD, catalase and Gpx activity, by over 50%, at a concentration of 200 mg/kg (Fig. 3a–c, *P *< 0.05). The GSH level decreased by 69.7% in the control rats. BLF treatment increased the GSH content by 69.6% and 169.5% at concentrations of 100 and 200 mg/kg, respectively (Fig. 3d, *P *< 0.05).

### Effect of BLF on total protein and total cells

**Fig. 4:**
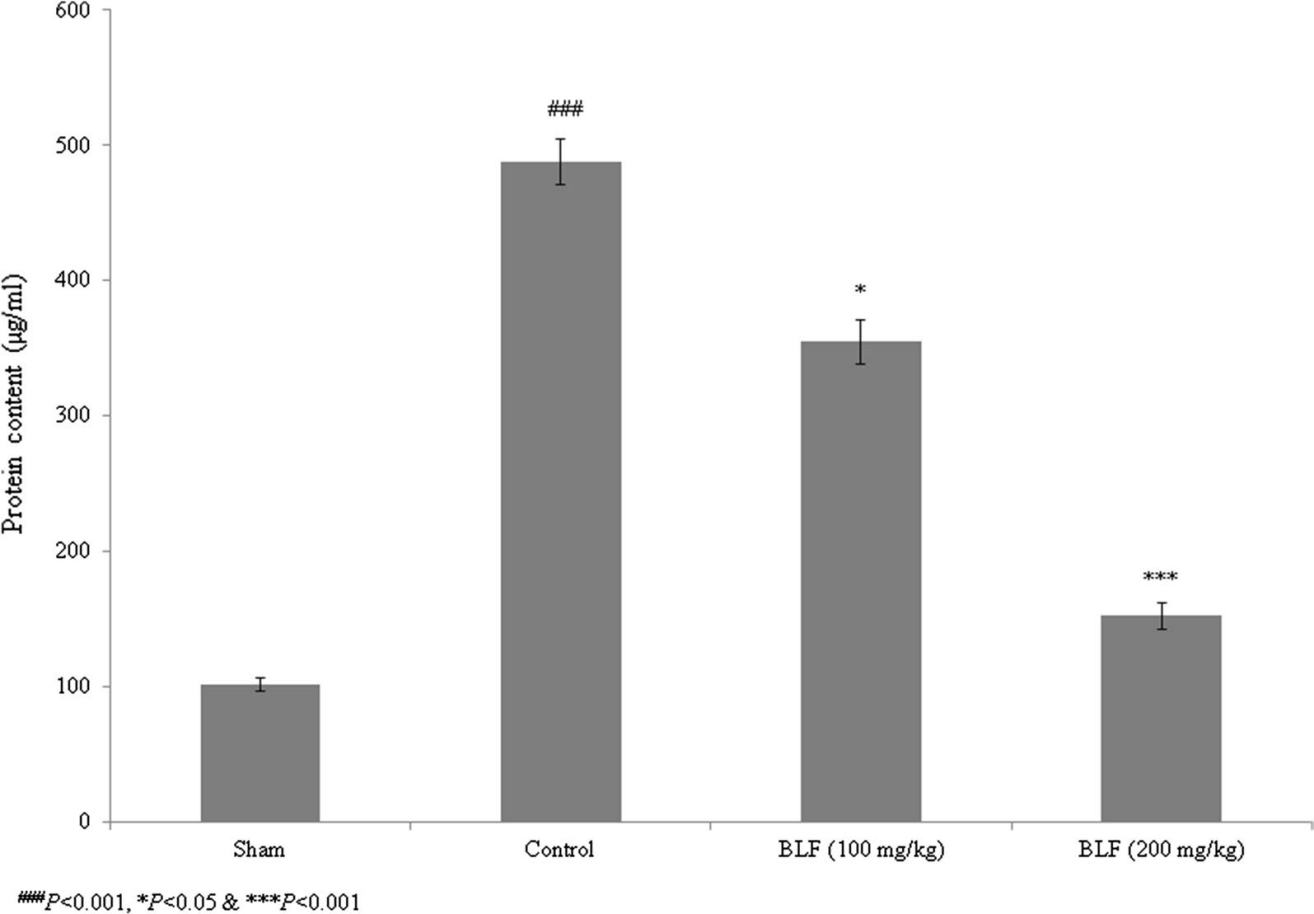
BLF reduces total protein content in the lung tissue homogenate of ALI-induced male albino rats. Rats were given BLF for 30 consecutive days through oral gavage. ^###^*P* < 0.001 compared to the sham group; *P < 0.05 and ***P < 0.001 compared to control rats

**Fig. 5:**
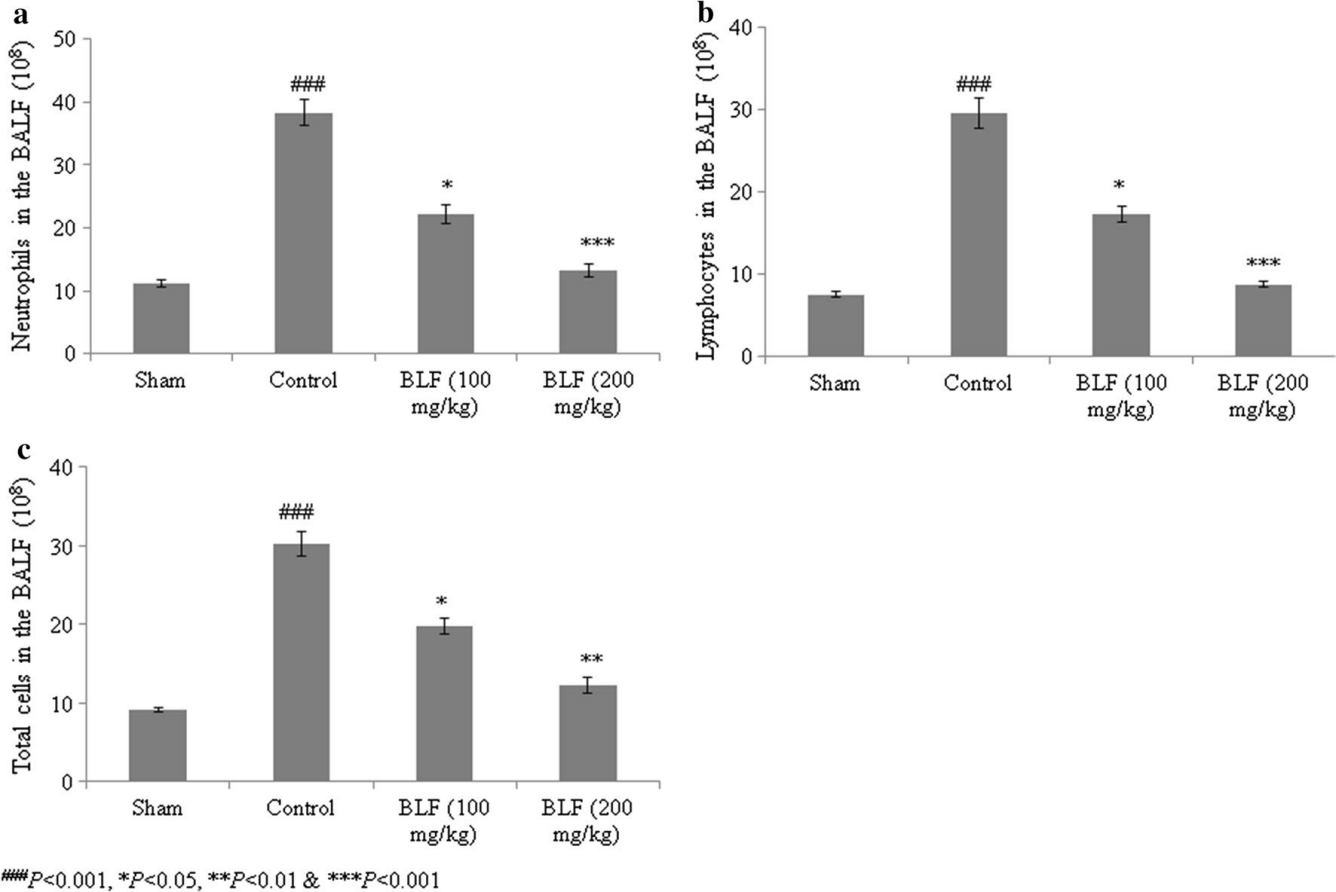
BLF reduces neutrophils (**a**), lymphocytes (**b**) and total cell count (**c**) in ALI-induced male albino rats. Rats were given BLF for 30 consecutive days through oral gavage. ^###^*P* < 0.001 compared to the sham group; *P < 0.05 and ***P < 0.001 compared to control rats

Total protein content increased by 381.3% in control rats compared to sham rats. BLF treatment reduced the total protein content by 27.2% and 68.7% at concentrations of 100 and 200 mg/kg, respectively (Fig. 4, *P *< 0.05). Lymphocytes, neutrophils and the total cell count increased substantially in control rats, but were reduced by more than 50% under BLF treatment at a concentration of 200 mg/kg (Fig. 5a–c, *P *< 0.05).

### Effect of BLF on inflammatory markers and myeloperoxidase

**Fig. 6:**
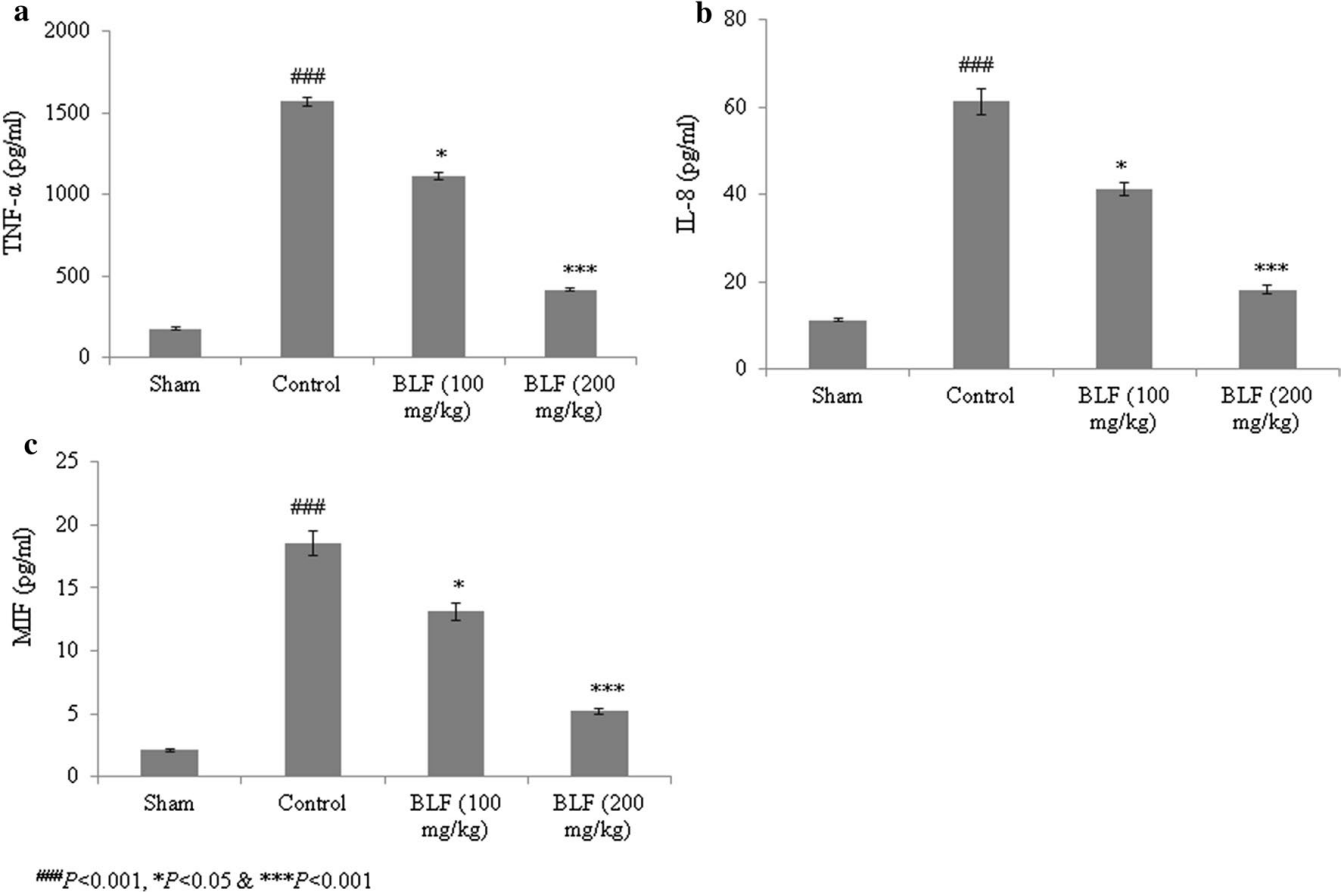
BLF reduces tumor necrosis factor-alpha (TNF-α) (**a**), interleukin-8 (IL-8) (**b**) and migration inhibitory factor (MIF) (**c**) levels in ALI-induced male albino rats. Rats were given BLF for 30 consecutive days through oral gavage. ^###^*P* < 0.001 compared to the sham group; *P < 0.05 and ***P < 0.001 compared to control rats

**Fig. 7:**
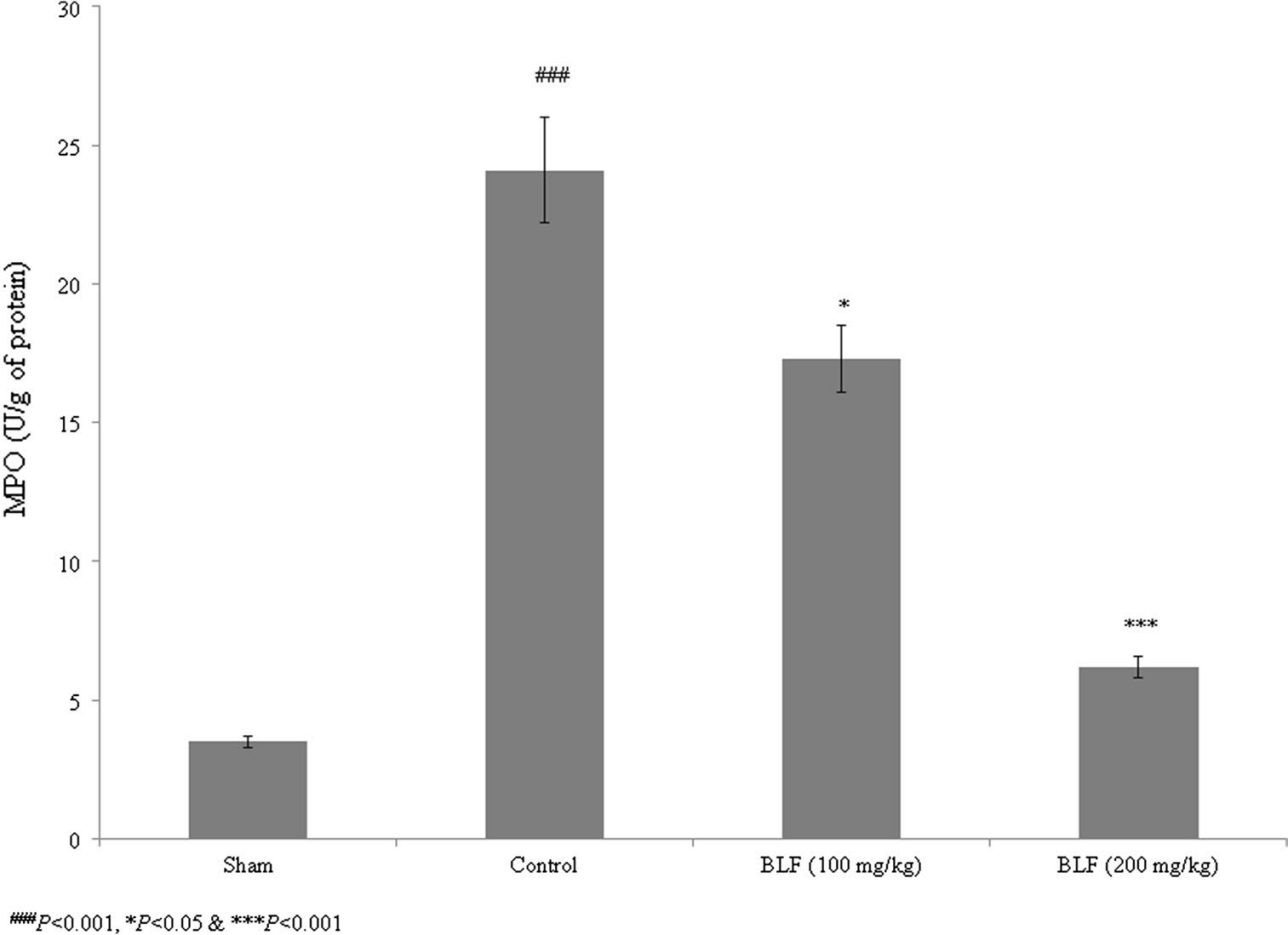
BLF reduces myeloperoxidase (MPO) activity in ALI-induced male albino rats. Rats were given BLF for 30 consecutive days through oral gavage. ^###^*P* < 0.001 compared to the sham group; *P < 0.05 and ***P < 0.001 compared to control rats

The TNF-α, IL-8 and MIF levels increased in control rats, but decreased by more than 50% under BLF treatment at a concentration of 200 mg/kg (Fig. 6a–c, *P *< 0.05). MPO activity was elevated by 588.6% in control rats relative to sham rats, but decreased by 28.2% and 74.3% under BLF treatment at concentrations of 100 and 200 mg/kg, respectively (Fig. 7, *P *< 0.05).

### Effect of BLF on cellular architecture of lung tissue

**Fig. 8:**
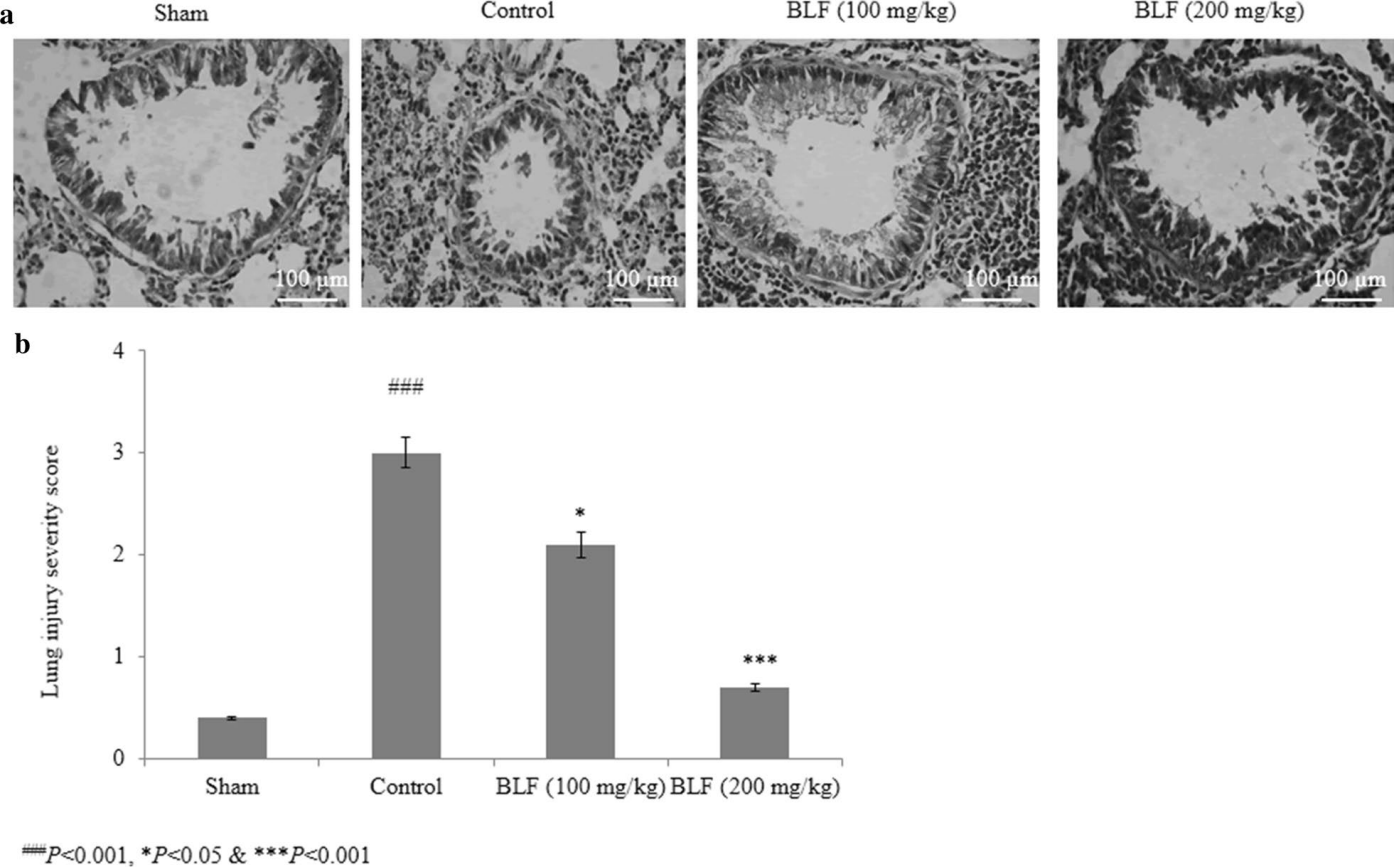
BLF restores intestinal microvilli damage to within the normal range and reduced inflammatory cell invasion in ALI-induced male albino rats. Rats were given BLF for 30 consecutive days through oral gavage. **a** Represents the histopathological images of lung tissues and **b** represents the lung injury severity score. ^###^*P* < 0.001 compared to the sham group; *P < 0.05 and ***P < 0.001 compared to control rats. Scale bar is 100 µm

Histopathological analysis of the sham rats showed normal cellular architecture. Neutrophilic infiltration and edema were found in the control rats (Fig. 8). However, BLF treatment restored intestinal microvilli injury to the normal range and reduced inflammatory cell invasion (Fig. 8).

## Discussion

We identified protective effects of BLF against sepsis-induced ALI in a rat model. Sepsis may lead to multiple organ failure and lung dysfunction (Fujishima 2016). Patients with ALI often present with tachypnea and hypoxemia (Randhawa and Bellingan 2007). ALI is associated with high rates of morbidity and mortality (Fang et al. 2012), and causes 74,500 deaths per year in Western countries Walkey et al. (2012). Increased permeability of the alveolar-capillary membrane, pulmonary edema, accumulation of protein-rich fluid in the airspaces, poor lung performance, and pulmonary infiltration of neutrophils are key symptoms of ALI (Matute-Bello et al. 2008). Favarin et al. (2013) reported that there is currently no cure for ALI, and that sepsis increases its mortality rate.

The destruction of the pulmonary endothelium and alveolar epithelium leads to lung dysfunction, which can result in complete failure of several organs in sepsis patients Ware and Matthay (2000). ILs, TNF-α, and several inflammatory cytokines and mediators, are produced from activated macrophages and neutrophils (Chen et al. 2017). In our study, neutrophils, lymphocytes, total cell count, IL-8, TNF-α and MIF substantially increased in control rats (ALI). However, 100 and 200 mg/kg of BLF treatment reduced these markers to within the normal range. Manzoni et al. (2009) reported that BLF prevents sepsis in low-birth-weight neonates, and Chen et al. (2014) demonstrated a therapeutic effect of aerosolized BLF against lung injury and fibrosis in mice. Cutone et al. (2019) reported that aerosolized BLF reduced infection, iron dysbalance and inflammation in chronic lung infection. In a rat model of acute kidney injury, Hegazy et al. (2016) identified renoprotective effects of BLF. Valenti et al. (2017) showed that aerosolized BLF reduces pro-inflammatory cytokines and neutrophils in a mouse model of lung infection.

The pathological importance of oxidative stress in ALI has been highlighted by previous studies (Kellner et al. 2017). Reactive oxygen species (ROS) react with macromolecules, leading to higher rates of lipid peroxidation (Kwiecien et al. 2014). Bhattacharyya et al. (2004) reported that neutrophils produce superoxide molecules during inflammation. In the present study, lipid peroxidation substantially increased in control rats (ALI). However, 100 and 200 mg/kg of BLF treatment reduced lipid peroxidation. This is consistent with the results of Faridvand et al. (2017) who showed that BLF treatment inhibited lipid peroxidation in rats. Collectively, these results suggest that BLF is an effective therapeutic agent against sepsis-induced ALI.

## Data Availability

Corresponding author could provide the all experimental data on valid request.
